# Knowledge and reported practices of men and women on maternal and child health in rural Guinea Bissau: a cross sectional survey

**DOI:** 10.1186/1471-2458-10-319

**Published:** 2010-06-08

**Authors:** Rebecca King, Vera Mann, Peter D Boone

**Affiliations:** 1 Effective Intervention, Centre for Economic Performance, London School of Economics, London, UK; 2 Medical Statistics Unit, Department of Epidemiology and Population Health, London School of Hygiene and Tropical Medicine, London, UK; 3 The Nuffield Centre for International Health and Development, Leeds Institute of Health Sciences, University of Leeds, Leeds, UK

## Abstract

**Background:**

Participatory health education interventions and/or community-based primary health care in remote regions can improve child survival. The most recent data from Guinea Bissau shows that the country ranks 5^th ^from bottom globally with an under-five mortality rate of 198 per 1000 live births in 2007. EPICS (Enabling Parents to Increase Child Survival) is a cluster randomised trial, which is currently running in rural areas of southern Guinea Bissau. It aims to evaluate whether an intervention package can generate a rapid and cost-effective reduction in under-five child mortality. The purpose of the study described here was to understand levels of knowledge on child health and treatment-seeking and preventative behaviours in southern Guinea Bissau in order to develop an effective health education component for the EPICS trial. The study also aimed to assess the effect of gender and ethnicity on knowledge and behaviour.

**Methods:**

Women and men were interviewed in their households using a structured questionnaire. Characteristics of the households and of the interviewed women and men were tabulated. The number of correct answers given to the health knowledge and practice questions and their percentage distribution were tabulated by items and by gender. An overall health knowledge score was derived.

**Results:**

There are low levels of appropriate knowledge on child health, some inappropriate practices and generally low vaccination coverage. Health knowledge scores improve significantly amongst those who have accessed higher education. Differences in health knowledge between women and men become insignificant once age and education are accounted for.

**Conclusions:**

Health education activities should be an integral part of a package to improve child survival in rural Guinea Bissau. These activities should focus on diarrhoea, malaria, pneumonia, pregnancy, delivery, neonatal care and vaccination coverage, as these are areas where knowledge and practices were found to be inadequate in this study. Men as well as women should be involved in these activities. Prior to developing health education interventions in similar settings, studies to assess areas to be targeted should be conducted.

## Background

Child survival came to the forefront of the global health agenda with the 2003 series in The Lancet [1-5]. The opening report stated that an estimated 10.8 million children aged under-five were dying globally in 2000. Approximately 90% of these deaths were occurring in 42 countries. The major causes of mortality were identified as diarrhoea and pneumonia, whilst neonatal disorders, malaria and HIV contributed substantially in certain regions [1]. An estimated 63% of these under-five deaths could be prevented if current knowledge on available and feasible interventions is translated into effective action [2]. One of the targets of the United Nations' Millennium Development Goals (MDGs) is to reduce the under-five mortality rate by two-thirds between 1990 and 2015 (MDG4). UNICEF, WHO and other experts agreed on a minimum set of key indicators for monitoring progress to achieve MDG4. These include oral rehydration therapy (ORT) and continued feeding for diarrhoea, the use of insecticide treated mosquito nets (ITNs) and antimalarial treatment for under-fives, antibiotic treatment for pneumonia, immunization coverage (tetanus, measles and DTP3), and timely initiation of breastfeeding and exclusive breast feeding until 6 months [6].

There is evidence to suggest that participatory health education interventions and/or community-based primary health care in remote regions can improve child survival [7,8]. These community-based interventions target health knowledge and promote appropriate treatment-seeking and preventative behaviours. In Nepal, an intervention using women's health groups led to a reduction in neonatal mortality of nearly 30%.These groups met monthly to identify problems and implement and evaluate strategies to tackle the identified problems [9]. A similar approach saw a significant decrease in perinatal mortality in Bolivia [10,11]. In India, home-based neonatal care and management of sepsis by trained community health workers led to a 62% decline in neonatal mortality when compared to a control region that surrounded the project area. Moreover, fatality from sepsis declined from 16.6% to 2.8% [12]. The evidence suggests that an intensive community health education programme in combination with mentoring of community health workers and improved availability of services and materials at the community level may lead to major, cost-effective declines in child mortality.

The most recent data ranks Guinea Bissau 5^th ^from the bottom globally. The neonatal mortality rate was 47 per 1000 live births in 2004 and the country had an under-five mortality rate of 198 per 1000 live births and an infant mortality rate of 118 per 1000 live births in 2007 [13]. There is very little information on the causes of child deaths in Guinea Bissau, as the country was not included in the epidemiological profiles supplied in the Child Survival series. However, nearby countries were included in Profile 2 and diarrhoea, malaria, pneumonia and neonatal disorders were identified as the leading causes of child mortality [1].

EPICS (Enabling Parents to Increase Child Survival) is a cluster randomised trial, which is currently running in rural areas of southern Guinea Bissau [14]. It aims to evaluate whether an intervention package can generate a rapid and cost-effective reduction in under-five child mortality. The package includes health education through health clubs and house-to-house visits. It also includes intensive training and supervision of village health workers and community-based birth attendants, improved outreach services and enhanced institutional delivery services. A maternal birth history survey conducted in the region before the trial started in 2007 found an under-five child mortality rate of 154 per 1000 live births, as reported by women aged 12 to 49 for their 2002 to 2007 pregnancy history. This is somewhat lower than nationally reported [13].

The aims of this study were to understand levels of knowledge on child health as well as the treatment-seeking and preventative behaviours of the inhabitants of the trial area. The study was conducted prior to the start of the trial in order to develop an appropriate and effective health education component. The study also aimed to assess the effect of gender and ethnicity on knowledge and behaviour.

This article reports on the knowledge and reported practices of men and women on maternal and child health in two districts of southern Guinea Bissau. It also reports the effect of gender and ethnicity of knowledge and behaviour.

## Methods

### Study setting and participants

The study was conducted in the two rural districts of southern Guinea Bissau (Quinara and Tombali) in which the EPICS trial is being implemented. Villages in the region were considered eligible for the trial if they were believed to have a population of approximately 300 inhabitants. The villages were mapped and enumerated during the maternal birth history baseline survey. Clusters were formed from one or more villages in order to create an average population of 350 per cluster. This process resulted in 241 villages being organised into 146 clusters for inclusion in the trial [14]. This baseline study of health knowledge and practice was conducted in 31 of these villages after the clusters had been identified but before they were randomised into control and intervention arms. The villages were selected on the basis of their accessibility (in the rainy season, when this study was conducted, some of the villages are inaccessible). The selected villages included those that had majority Fula (13), Beafada (10) and Nalu (5) populations. These are ethnic groups that normally identify themselves as Muslim. Two villages had Susu populations and one had Balanta population as majority.

In each selected village, 10 households out of those that had already been mapped were randomly selected for this study. Within each household, one woman who was a primary carer of a child aged five or under and one man who lived in the same household as the woman (most commonly her husband) were selected for interview. If a visited household had more than one eligible woman and/or man, the ones who were interviewed were randomly selected.

### Data collection and instruments

A fully structured questionnaire (Additional file 1) was developed and interviews were carried out by trained field workers who were supervised. The questionnaire -- which was in part based on questions from DHS surveys - collected information on knowledge and reported practices on the key indicators affecting child survival [3]. It was arranged into seven sections on household characteristics, respondent characteristics, health knowledge, pregnancy and delivery, vaccination status, treatment-seeking behaviour and household observation. Section one was completed with the woman and man together. Sections two, three and parts of six were completed with both the woman and man, interviewed separately. Sections four, five, parts of six and seven were completed only with the woman. Sections one and two had questions with only one possible answer. The other sections had two types of question: those for which only one answer could be given and those for which more than one answer was possible.

The questionnaire was piloted in two different villages in southern Guinea Bissau and revisions were made before the survey was conducted. These villages were excluded from the survey. A team of six field workers -- recruited from Bissau - and one field supervisor were provided with intensive classroom and field-based training as well as a detailed manual of survey guidelines. Field workers conducted the survey in Kriol or, where necessary, directly or through translation in appropriate local languages.

### Data analysis

Characteristics of the households and of the interviewed women and men were tabulated. These included background characteristics of the households and demographic characteristics of the respondents by gender. Descriptive statistics for continuous variables included the mean, standard deviation, median, and range and for categorical variables the numbers and percentages. The number of correct answers given to the health knowledge and practice questions and their percentage distribution were tabulated by items and by gender (as appropriate).

An overall health knowledge score was derived by summing the total number of correct responses given to the fifteen health knowledge questions in section three of the questionnaire. Questions with more than one possible correct answer were weighted by the inverse of the total number of correct answers so that no single question contributed more than one point out of fifteen to the score. Factors affecting health knowledge scores were investigated using simple linear regression models. Gender was used as a single binary predictor. Education was categorised into primary (grade 1-6), secondary (grade 7-9), higher (grade 10-12) or Koranic education. Ethnicity was included in the models as Balanta, Beafada, Fula, Nalu, Mandinka, Susu and Bijagos, or other. Age and an indicator of household crowdedness (the number of people per room within the house) were used as continuous variables. Robust standard errors were used to allow for clustering within villages. The final multiple linear regression includes gender and ethnicity and other factors which either had a statistically significant effect on obtained health knowledge score themselves or confounded the effect of gender and/or ethnicity.

### Ethical considerations

This study was approved by the ethics committees of 'The Centre for Coordination of the Research of the Ministry Of Health' in Guinea Bissau (NCP-021/2007) and the London School of Hygiene and Tropical Medicine (5173).

## Results

### Descriptive statistics

The general characteristics of the 310 households are presented in Table 1. An average of 11 people and 3 children aged under-five lived in each household. An average of 2.5 people shared a room. This table also includes fieldworkers' observations on household facilities. In 295 households (95%) a bed net was observed in the place where it was said the youngest child sleeps (data not in table). 82% of households had a hand washing facility close to where the food is prepared, but only 17% had soap to wash the hands with. 81% of the households had a latrine.

**Table 1:** Household characteristics

	min	max	mean	SD	median
**Interviewee responses**					

Number of people living in household	3	34	10.5	5.7	9

Number of rooms in household	1	12	4.6	2.1	4

Number of people per room	0.7	12	2.5	1.3	2.3

Number of children living in household	1	12	2.7	1.7	2

Number of bed nets in household	0	13	3.6	2.2	3

	Number of households	%			

Time since nets were treated					

Not needed or within 6 months	49	15.8			

Within 1 year	153	49.4			

Never/Don't know	102	32.9			

No bed nets	6	1.9			

**Interviewer observations**	Number of households	%			

Hand washing facility where food is prepared					

Yes	253	81.6			

Soap to wash hands Yes		17.1			

Latrine type No latrine	58	18.7			

Clean*	185	59.7			

Covered*	133	42.9			

Hand washing facility close to latrine*	8	2.6			

*More than one can apply

Table 2 presents the demographic characteristics of women and men interviewed for this study as well as their education and literacy level. In 216 households the interviewed man was the husband of the woman (data not shown in the table). On average, women were much younger than men (mean age of 27.9 years versus 40.7 years for females and males respectively). The majority of respondents were Fula or Beafada. The distribution of women's ethnicity was similar to men. On average, women had lower education levels than men and 65% of women had never attended school compared to 18% of men. Eighty three percent of women and 29% of men could not read the sample sentence.

**Table 2:** Demographic characteristics of interviewed women and men

	Women		Men	
	**number**	**%**	**number**	**%**

Age (year, mean, SD)	27.9	7.7	40.7	14.5

Ethnicity				

Balanta	15	4.8	15	4.8

Beafada	99	31.9	97	31.3

Fula	133	42.9	132	42.6

Nalu	32	10.3	34	11.0

Mandinka	12	3.9	12	3.9

Susu	7	2.3	7	2.3

Other	12	3.9	13	4.2

Never attended school	202	65.2	56	18.1

Education level				

Primary	85	27.4	116	37.4

Secondary	4	1.3	30	9.7

Higher	0	0.0	10	3.2

Koranic	19	6.1	98	31.6

Can't read	257	82.9	90	29.0

### Health knowledge

Table 3 summarises the health knowledge of the interviewees by gender for each of the questions asked. For questions in which only one answer could be given, the number and percentage of interviewees who gave the correct answer is shown. The number and percentage of interviewees giving various numbers of correct answers are provided in the table for questions with more than one correct answer. Knowledge on the prevention and management of diarrhoea was low. Around half of respondents knew that a child with diarrhoea needs to drink more fluids than normal. About the same number recognised a packet of oral rehydration salt (ORS) when it was shown to them. However, only 34% of women and 36% of men knew that a baby aged under six months who has diarrhoea should be given breast milk followed by ORS. Likewise, knowledge on the prevention of malaria was low and 46% of women and 68% of men knew that malaria is spread through mosquito bites. Over 56% of women and 44% of men could not name any antimalarials that are used to treat malaria. Only 21% of women and 33% of men knew that a child taking chloroquine for malaria should be treated for three days, even if they feel better (i.e. they must complete treatment). Very few women (12%) and men (29%) stated that they had ever heard the term (pneumonia) and only one woman could give a specific symptom of pneumonia. Nobody knew about the use of antibiotics for treating pneumonia. Whilst the majority of respondents had heard of HIV/AIDs, very few knew that measles can be prevented by vaccination.

**Table 3:** Health knowledge

Questionnaire items (number of possible correct answers)	Women (N = 310)		Men (N = 310)	
	**Number**	**%**	**Number**	**%**

Number of correct answers given to prevent diarrhoea (4)				

0	222	71.6	194	62.6

1	41	13.2	39	12.6

2	45	14.5	64	20.6

3	2	0.6	13	4.2

4	0	0.0	0	0.0

Number said to give more drink to child with diarrhoea	152	49.0	161	51.9

Number who know ORS	148	47.7	144	46.5

Number said to give breast milk followed by ORS if necessary to a baby under 6 months with diarrhoea	105	33.9	112	36.1

Number said to dress lightly and cool child with damp cloth if child is sick with fever	249	80.3	242	78.1

Number heard about malaria	277	89.4	301	97.1

Number said people become infected with malaria from mosquito bite	141	45.5	212	68.4

Number of correct answers given to avoid malaria (4)				

0	169	54.5	91	29.4

1	80	25.8	123	39.7

2	57	18.4	84	27.1

3	4	1.3	12	3.9

4	0	0.0	0	0.0

Number of correct medicines to treat malaria (4)				

0	174	56.1	137	44.2

1	127	41.0	156	50.3

2	9	2.9	17	5.5

3	0	0.0	0	0.0

4	0	0.0	0	0.0

Number said to give chloroquine to a child with malaria at least for 3 days even if the child gets better	66	21.3	103	33.2

Number heard about pneumonia	36	11.6	90	29.0

Number of correct answers given for signs of pneumonia (3)				

0	309	99.9	310	100.0

1	1	0.003	0	0.0

Number said that antibiotics are treatment for pneumonia	0	0.0	0	0.0

Number said vaccine is the correct way to avoid measles	61	19.7	65	21.0

Number who heard about HIV/AIDS	280	90.3	303	97.7

Simple linear regressions (Table 4) showed that women on average scored one point less on health knowledge than men (p < 0.001), with an overall mean of 5.60 (SD = 0.14) and 6.58 (SD = 0.14) respectively. People with higher levels of education achieved substantially higher scores (p < 0.0001). Although the number of people living in each room in a household reduced the knowledge score, this reduction was neither substantial nor statistically significant (p = 0.15). The highest knowledge scores were achieved by Nalu (66 respondents) and Balanta (30 respondents), however, the effect of ethnicity was not statistically significant (p = 0.8). With each year increase in age the score is increased by 0.03 (p = 0.003). Multiple regressions revealed that the apparently lower knowledge score for women was the result of the confounding effect of education level. In the final model, once the effect of gender is controlled for age, education level and ethnicity, women score 0.1 points higher than men and the effect of gender becomes statistically insignificant (p = 0.7). The effect of age and education level estimated in this final model did not materially change compared to their effects in the simple regressions. The effect of ethnicity remained statistically insignificant in the multiple regression.

**Table 4:** Linear regressions for health knowledge score (adjusted for clustering in villages)

	number*	Univariable models			Multivariable model		
**variable (unit)**		change in score**	**95% CI**		change in score**	**95% CI**	

woman vs man	-	-0.97	-1.29	-0.66	0.09	-0.37	0.56

age (year)	-	0.03	0.01	0.05	0.03	0.008	0.05

education primary vs none							

primary vs none	201	1.69	1.28	2.11	1.66	1.17	2.14

secondary vs none	34	2.83	2.09	3.57	2.90	2.07	3.73

higher vs none	10	4.78	3.87	5.69	4.91	3.89	5.93

koranic vs none	117	0.60	0.07	1.13	0.53	-0.15	1.21

crowdedness (person/room)	-	-0.16	-0.38	0.06	-	-	-

ethnicity							

beafada vs balanta	196	-0.11	-1.30	1.08	0.05	-1.06	1.17

fula vs balanta	265	-0.24	-1.39	0.92	-0.24	-1.31	0.82

nalu vs balanta	66	0.20	-1.08	1.48	-0.13	-1.28	1.01

mandinka vs balanta	24	-0.88	-2.78	1.03	-0.70	-2.29	0.89

susu vs balanta	14	-0.69	-3.18	1.81	-0.79	-2.38	0.81

other vs balanta	25	-0.45	-1.79	0.88	-0.81	-2.07	0.45

* number of the 620 respondents in the given category

** change in the average health knowledge score for a unit increase in the continuous variables or being in a category versus the baseline category for categorical variables.

Mothers were also asked questions on exclusive breastfeeding and risk signs during pregnancy. Thirty two percent knew that complementary feeding should start at 6 months and 66% could not identify any risk signs.

### Pregnancy and delivery

Ninety three percent of women stated that they had received antenatal care (Table 5). The majority of these checkups were conducted in clinics or hospitals (88%). Most deliveries occurred at home with only around 23% in a health facility. Health professionals attended 24.4% of deliveries. In 68% of deliveries, a razorblade was used to cut the umbilical cord and 79% of women stated that the equipment used was sterilised. The umbilical cord was reported to be cleaned with alcohol by 17% of women. Only 25% of women reported commencing breastfeeding immediately or within one hour of the birth.

**Table 5:** Antenatal care and delivery practices (from the most recent pregnancy)

	Number	% of women
Number of women received at all antenatal care	288	92.9

Number of antenatal checkups		

0	22	7.1

1-3	74	23.9

4-6	134	43.2

7-9	55	17.7

Other	6	1.9

Do not know	19	6.1

Place where checkups happened*		

At home by nurse	2	0.6

USB	33	10.6

regional clinic/NG clinic/hospital	273	88.1

Not specified	6	1.9

Place of delivery		

Husband home	163	52.6

Parent's home	53	17.1

Someone else's home	17	5.5

USB	6	1.9

Regional clinic	55	17.7

Hospital	9	2.9

Not specified	7	2.3

Attendance at delivery**		

Doctor	6	1.9

Nurse	28	9.0

Midwife	42	13.5

Matrona	74	23.9

Relative	141	45.5

Friend	16	5.2

Not specified	20	6.5

No one	8	2.6

Equipment used to cut umbilical cord		

Razorblade	210	67.7

Household knife	7	2.3

Scissors	51	16.5

Other	2	0.6

Do not know	40	12.9

Equipment was sterilised		

No	3	1.0

Yes	246	79.4

Do not know	61	19.7

Equipment sterilisation method		

New blade	215	69.4

Alcohol	12	3.9

Heat	9	2.9

Washing with soap	3	1.0

Other	2	0.6

Do not know	6	1.9

Umbilical cord dressing ***		

Alcohol	53	17.1

Palm Oil	74	23.9

Siti malagos	3	1.0

Bandage	54	17.4

Nothing	21	6.8

Other	106	34.2

Do not know	42	13.5

Time to put baby on breast after birth		

Immediately or within 1 hour	78	25.2

Within 24 hours	156	50.3

Within 3 days	67	21.6

Within 1 week	7	2.3

Within 1 month	2	0.6

*a woman could have checkups at more than one place

**more than one type of attendance could be reported

***more than one dressing could be reported

### Vaccination coverage

Vaccination coverage for the youngest child of each of the women interviewed is summarised in Table 6. Of the 310 women only 245 had a vaccination card for their youngest child, thus the analysis could only be done for these children. 88% of children aged one week and over had received their BCG vaccination. Around 90% of children aged 6 weeks and over had received their OPV1 and DPT1 vaccinations. Vaccination coverage falls as the children get older. 56% of children who had a vaccination card received vaccinations in regional clinics.

**Table 6:** Vaccination coverage (from the vaccination card of the youngest child)

age of youngest	number	Vaccine	vaccinated		not vaccinated		card unreadable	
**child**	**with card**		**number**	**%**	**number**	**%**	**number**	**%**

≥1 week	245	BCG	215	87.8	27	11.0	3	1.2

≥6 weeks	240	OPV1	218	90.8	18	7.5	4	1.7

≥10 weeks	239	OPV2	175	73.2	62	25.9	2	0.8

≥14 weeks	234	OPV3	146	62.4	86	36.8	2	0.8

≥6 weeks	240	DPT1	216	90.0	21	8.8	3	1.2

≥10 weeks	239	DPT2	175	73.2	63	26.4	1	0.4

≥14 weeks	234	DPT3	154	65.8	79	33.8	1	0.4

≥9 months	194	Measles	149	76.8	43	22.2	2	1.03

### Treatment-seeking behaviour

Treatment-seeking behaviour of women and men was quite different. Fifty percent of women stated that they would seek help from another parent if a child was sick. In contrast, 60% of men stated they would seek help from a clinic or a hospital if a child was sick (Table 7). Men and women gave similar reports for the places where medicines for children are normally bought.

**Table 7:** Treatment-seeking behaviour

	Women (N = 310)		Men (N = 310)	
	**Number**	**%**	**Number**	**%**

Person from whom help is sought if child is ill				

Another Parent	156	50.3	14	4.5

Relative	40	12.9	68	21.9

Traditional Healer	3	1.0	12	3.9

Asc	7	2.3	13	4.2

Matrona	2	0.7	1	0.3

Clinic	84	27.1	180	58.1

Hospital	1	0.3	6	1.9

Other	17	5.5	16	5.2

Where is medicine bought normally*				

Local Person	59	19.0	56	18.1

Relative	2	0.6	0	0.0

Pharmacy	111	35.8	130	41.9

USB	23	7.4	29	9.4

Local Clinic	182	58.7	162	52.3

Other	46	14.8	28	9.0

Do Not Know	0	0.0	1	0.3

Which of the listed medicine kept at home*				

ORS	4	1.3	2	0.6

Chloroquine and/or Fansidar	15	4.8	19	6.1

Paracetamol	16	5.2	25	8.1

Bactrim	1	0.3	9	2.9

Amoxycilin	1	0.3	3	1.0

Traditional Medicine	9	2.9	25	8.1

Other	23	7.4	40	12.9

Cannot Identify	9	2.9	14	4.5

None	254	81.9	225	72.6

*more than one answer could be given

Women were also asked whether they can decide by themselves whether or not a seriously ill child should be taken for medical treatment and 71% stated that they can (data not shown in table).

## Discussion

The study found low levels of appropriate knowledge on child health, some inappropriate practices and generally low vaccination coverage. The study showed that health knowledge scores improve significantly amongst those who have accessed education, in line with other studies [15,16]. Regression analysis revealed that differences in health knowledge between women and men become insignificant once age and education are accounted for.

Most of the results presented in this paper are in line with the UNICEF findings on Guinea Bissau in their 2008 report, which tracks the key indicators for monitoring progress to achieve MDG 4 [17]. Any differences might be explained by the fact that part of this study focused on knowledge, whereas the UNICEF report details reported practice. Furthermore, WHO and UNICEF estimate immunisation coverage of 89% for BCG, 63% for 3 doses of DPT, 64% for 3 doses of polio (OPV), and 76% for measles in 2007 [18]. This is very close to the findings of this study of 88%, 66%, 62% and 77% respectively for the same year.

This study has some limitations. First, the findings of this survey on levels of health knowledge and treatment-seeking and preventative behaviour are only representative of the area selected for the EPICS trial rather than the population of southern Guinea Bissau as a whole. Second, the findings could be biased due to the constraints of conducting research in the difficult conditions of the rainy season. Villages were selected for this survey on the basis of their accessibility, rather than randomly. This resulted in higher numbers of Fula (43%), lower numbers of Beafada (31%) and very low numbers of Balanta (5%) respondents compared to the representation of these groups within the population, based on the enumeration data for the EPICS trial on 7876 women (Beafada (35%), Fula (17%) and Balanta (21%)). This is probably due to the fact that the clustered settlements in which the Beafada and Fula most commonly live are often found in more accessible on-road locations, whereas the scattered settlements in which the Balanta most commonly live are often found further away from the main roads.

Despite its limitations, the method employed in this study has some useful implications for future research. Very few studies of health knowledge and behaviour bring together the range of issues covered in this survey. The majority of studies were conducted on mothers or women of reproductive age on single themes such as the prevention and management or treatment of diarrhoea [19,20], malaria [21,22], and pneumonia [23,24], as well as on knowledge and practice surrounding antenatal care, delivery [25,26] and breastfeeding [27,28]. There are a number of studies on health knowledge -- particularly on malaria - which were conducted on both women and men (parents, caregivers, or community members in general) [29-31]. Of the studies mentioned above, many were conducted at the facility level and/or in urban settings and very few studies compared female and male knowledge and practice on a range of issues related to child health at the household level in rural settings. An exception is a publication, which examined community effects such as the proportion of literate adults and the presence of a market, on health knowledge in Ghana [15]. As integrated approaches to the reduction of child mortality at the community level become more widespread, it becomes critical to develop tools that can capture a range of knowledge and practice of women and men at the household level in rural settings in order to plan appropriate and effective interventions. The authors believe the survey conducted in this study is a useful contribution to this.

Furthermore, most studies of health knowledge that have been conducted in rural areas of developing countries have reported only descriptive statistics, the numbers and/or percentages of respondents giving correct answers or all the possible correct answers for each individual health knowledge related question, or they derived a simple scoring system which increases by one for each correct answer [32,33,24]. In this study, an overall health knowledge score, which is weighted by the inverse of the possible correct answers, has been developed. The authors have used regression analyses, which take into account the clustering of the sample in the villages by using robust standard error estimates, to assess the association between factors and overall health knowledge. As far as the authors are aware, only one group has allowed for hierarchical structure in their analyses [15]. This approach creates new possibilities for measuring changes in health knowledge on multiple issues over time.

Finally, one of the aims of this study was to gather information to inform the development of an appropriate and effective health education programme in southern Guinea Bissau. The study provided critical information for programme planning both in the short and long term. In the short term, targeted programmes have been developed that address key gaps in health knowledge and practice, as outlined by the findings of this survey. These programmes primarily target women as the primary carers of children aged under-five, but they also include men who, as this survey shows, are frequently referred to by their wives when a child is sick. Specifically, the health club methodology [34] has been adapted to address health knowledge and practice in relation to child survival. Health clubs are community associations that hold weekly meetings to discuss health issues in a highly structured participatory framework. This survey provided critical information to develop the content of the health clubs for the intervention (EPICS trial) in southern Guinea Bissau. In the long term, programmes must be developed that address the chronic lack of access to education in the region, particularly amongst women.

## Conclusion

Prior to planning community-based health education interventions, it is important to conduct properly designed surveys to assess levels of knowledge on child health, treatment-seeking and preventative practices as well as vaccination coverage.

This study found low levels of appropriate health knowledge on diarrhoea, malaria and pneumonia as well as on breastfeeding and risk signs during pregnancy. It also found that the majority of births occurred at home without the presence of a skilled birth attendant and that poor practices existed in relation to the care of the umbilical cord and immediate breastfeeding. The results also showed inappropriate levels of vaccination coverage. Therefore, health education activities that target these areas of knowledge and practice should be an integral part of an intervention package to improve child survival in rural Guinea Bissau.

Although child health education activities often target women only, our study in rural Guinea Bissau showed it could be crucial to involve men in health education activities to improve child survival.

Finally, the derivation of a weighted overall health knowledge score and its use in robust regression analysis is addressing an important methodological issue in assessing health knowledge on multiple topics.

## Competing interests

The authors declare that they have no competing interests.

## Authors' contributions

RK contributed to the conception and design of the study, the acquisition of data, the interpretation of the data, and the drafting of the manuscript. VM contributed to the conception and design of the study, the analysis and interpretation of the data, and the drafting of the manuscript. PB contributed to the conception and design of the study and the revision of the manuscript. All authors read and approved the final manuscript.

## Pre-publication history

The pre-publication history for this paper can be accessed here:

http://www.biomedcentral.com/1471-2458/10/319/prepub

## Supplementary Material

Additional file 1 **Health knowledge baseline questionnaire**. This file contains the survey questionnaire that was administered in this study. Click here for file
