# Supplementary material for: Knowledge and reported practices of men and women on maternal and child health in rural Guinea Bissau: a cross sectional survey
Source: BMC Public Health. 2010 Jun 8;10:319. doi: 10.1186/1471-2458-10-319 (PMC2891651; doi:10.1186/1471-2458-10-319)
Supplement: Additional file 1 — Health knowledge baseline questionnaire. This file contains the survey questionnaire that was administered in this study. [file 1471-2458-10-319-S1.DOC]

**EFFECTIVE INTERVENTION**

**MATERNAL AND CHILD HEALTH IN GUINEA BISSAU**

**HEALTH KNOWLEDGE AND BEHAVIOUR BASELINE SURVEY**

**JULY 2007**

**Please give the following information:**

My name is____________________. I work with Effective Intervention. You might have met some of my colleagues who have already conducted surveys in Quinara and Tombali.

Effective Intervention is an NGO that implements programmes that are related to child health. We will be working in selected *tabancas* in the region from October 2007. In order to create programmes that are relevant to the people in Quinara and Tombali, we would like to better understand the things that they know about child health issues.

Therefore, I would like to ask you to participate in an interview. For women, the interview will take around 45 minutes. For men, the interview will take around 15 minutes. Your participation is voluntary. If you do not wish to take part, please tell me now. If you do wish to take part then you can refuse to answer any questions and you can stop the interview at any time. You will not receive anything in exchange for taking part in this interview.

I will keep your responses confidential. Only researchers involved in this study will look at the findings.

Please do not be concerned if you do not know the answers to any questions. We are here to understand what people know and do not know! Please be comfortable and answer the questions in your own time.

Do you have any questions?

| **Identification** | | | |
| --- | --- | --- | --- |
| Tabanca Number:  **Copy from the list** |  | | |
| Tabanca Name:  **Write the name in capital letters.** | ______________________________________________________ | | |
| Household Number:  **Copy from the house** |  | | |
| Woman’s Name:  **Write the full name in capital letters!** | ______________________________________________________ | | |
| Man’s Name:  **Write the full name in capital letters!** | _______________________________________________________ | | |
| Relationship of Man to Woman  **Circle one** | 1=Husband  2=Father  3=Father-in-law  4=Brother-in-law  5=Son  6=Brother  7=Other Relation  8=Not related | | |
| Interview Conducted By:  **Write your name in capital letters** | _______________________________________________________ | | |
| Date:  **Write today’s date** | _______________________________________________________ | | |
| Time Started:  **Write the start time** |  | Time Finished:  **Write the finish time** |  |

**Explain that the first section relates to the household of the respondents:**

| **Section 1: Household Information** | | |
| --- | --- | --- |
| **1.1** | How many people live in your household?  **Probe to include all adults, children and babies.**  **Write the number in the boxes.**  *Note: A household is defined as a group of people who live under one roof.* |  |
| **1.2** | How many rooms are there in your house (or the section of the building that your household occupies)?  **Include all separate rooms. Do not include outdoor kitchens or outdoor latrines.**  **Write the number in the boxes.** |  |
| **1.3** | How many children under the age of five live and sleep in your house (or the section of the building that your household occupies) every day?  **Write the number in the boxes.** |  |

**Now, explain that you need to interview the woman and the man separately. Request that the man waits somewhere nearby, but where he cannot hear the answers to the questions!**

**WOMAN’S SURVEY**

**Explain to the woman that the first section relates to information about her.**

| **Section 2: Respondent Information** | | |
| --- | --- | --- |
| **2.1** | Sex  **Circle one** | 1=Male  2=Female |
| **2.2** | Age  **Ask how old she is in years.**  **Write the age in the boxes** |  |
| **2.3** | Ethnicity  **Ask her which ethnic group she belongs to.**  **Circle one** | 1=Balanta  2=Biafada  3=Fula  4=Nalu  5=Mandinka  6=Susu  7=Bijagos  8=Other |
| **2.4** | Schooling  **Ask if she has ever attended school.**  **Circle one** | 0-No  1=Yes |
| **2.5** | Education Level  **If yes, ask her what is the highest level she attended or is attending.**  **If the answer to 2.4 is no, circle Not Applicable**  **Circle one** | 1=Primary (Grade 1-6)  2=Secondary (Grade 7-9)  3=Higher (Grade 10-12)  4=Koranic School  5=Not applicable |
| **2.6** | Years of Schooling  **If yes, ask her how many years she attended school in total.**  **Write the number.**  **If the answer to 2.4 is no, write N A for Not Applicable** |  |
| **2.7** | Literacy Level  **Ask her to read the sentence. If she cannot read the whole sentence, probe to read part of the sentence.**  **Circle one** | 1=Cannot read at all  2=Able to read only part of the sentence  3=Able to read the whole sentence  4=No appropriate language card available |

**Explain that the following questions relate to health knowledge.**

| **Section 3: Health Knowledge** | | |
| --- | --- | --- |
| **3.1** | If a child has diarrhoea, should he or she be given less to drink than usual, the same amount to drink as usual or more to drink than usual?  **Circle one** | 1=Less than usual  2=The same as usual  3=More than usual  4=Don’t know |
| **3.2** | Do you know any ways to prevent diarrhoea?  **Circle all that are mentioned** | A=Washing hands  B=Drinking clean water  C=Eating clean food  D=Using latrines  E= Putting lemon or lime in water  F=Other  G=Don’t know |
| **3.3** | Show her a packet or ORS.  **Ask, have you ever seen this before?**  **Circle one** | 0=No  1=Yes |
| **3.4** | Should a baby under the age of six months who has diarrhoea be given only breast milk, breast milk followed by ORS if necessary, only ORS, ORS followed by breast milk if necessary, nothing at all, or none of the above?  **Circle one** | 1=Only breast milk  2=Breast milk, followed by ORS if necessary  3=Only ORS  4=ORS, followed by breast milk if necessary  5=Nothing  6=None of the above  7=Don’t know |
| **3.5** | If a child is sick with fever, should he or she be wrapped up warmly and placed in a warm place, dressed as normal, dressed lightly and cooled with damp cloths, or none of the above?  **Circle one** | 1=Wrapped up warmly and placed in a warm place  2=Dressed as usual  3=Dressed lightly and cooled with damp cloths  4=None of the above  5=Don’t know |
| **3.6** | Have you ever heard of malaria?  **Circle one** | 0=No  1=Yes |
| **3.7** | If yes, how do people become infected with malaria?  **Circle all that are mentioned**  **If the answer to 3.6 is no, circle Not Applicable** | A=From a mosquito bite  B=Other  C=Don’t Know  D=Not applicable |
| **3.8** | How can people avoid malaria?  **Circle all that are mentioned**  **If the answer to 3.6 is no, circle Not Applicable** | A=Sleep under a bed net  B=Use insect repellents  C=Cover skin in the evening  D=Clear mosquito breeding sites (standing water; Vegetation)  E=Other  F=Don’t know  G=Not applicable |
| **3.9** | What medicines can be used to treat malaria?  **Circle all that are mentioned**  **If the answer to 3.6 is no, circle Not Applicable** | A=Chloroquine  B=Fansidar  C=ACT  D=Quinine  E=Paracetomol  F=Aspirin  G=Traditional medicines  H=Other  I=Don’t know  J=Not applicable |
| **3.10** | If you use chloroquine to treat a child with malaria should you give it once and then stop, keep giving it until the child feels better and then stop, give it for at least three days even if the child feels better, or something else?  **Circle one** | 1=Once, then stop.  2=Keep giving it until the child feels better, then stop.  3=Give it for at least three days even if the child feels better.  4=Something else  5=Don’t know  6=Not applicable |
| **3.11** | Have you ever heard of pneumonia?  **Circle one** | 0=No  1=Yes |
| **3.12** | If yes, what are the key symptoms of pneumonia?  **Circle all that are mentioned**  **If the answer to 3.11 is no, circle Not Applicable** | A=Cough  B=Breathing difficulties (fast or noisy breathing)  C=Indrawn chest  D=Other  E=Don’t know  F=Not applicable |
| **3.13** | If a child has pneumonia, what are the possible treatments that could cure the child?  **Circle all that are mentioned**  **If the answer to 3.11 is no, circle Not Applicable** | A=Antibiotics  B=Traditional medicines  C=Other  D=Don’t know  E=Not applicable |
| **3.14** | What is the best way to prevent measles?  **Circle all that are mentioned** | A=Vaccine  B=Keep children away from infected children  C=Other  D=Don’t know |
| **3.15** | Have you ever heard of HIV / AIDS?  **Circle one** | 0=No  1=Yes |

**Explain that the following questions relate to pregnancy and antenatal care.**

| **Section 4: Pregnancy and Antenatal Care** | | |
| --- | --- | --- |
| **4.1** | During your last pregnancy, did you receive antenatal care?  **Circle one** | 0=No  1=Yes |
| **4.2** | If yes, how many check ups did you receive?  **Circle one**  **If the answer to 4.1 is no, circle Not Applicable** | 1=1  2=2  3=3  4=4  5=5  6=6  7=7  8=8  9=9  10=Other  11=Don’t know  12=Not applicable |
| **4.3** | Where did you receive the check-ups?  **Circle all that are mentioned**  **If the answer to 4.1 is no, circle Not Applicable** | A=Nurse came to the *tabanca*  B=USB  C=Regional Clinic  D=Hospital  E=Non-governmental clinic or hospital  F=Elsewhere  G=Not applicable |
| **4.4** | Where did the delivery occur?  **If the woman says that the delivery began in one location and that she was moved during the delivery, circle the number that corresponds to the place where the baby was actually born.**  **Circle one** | 1=Husband’s home  2=Parent’s home  3=Someone else’s home  4=USB  5=Regional Clinic  6=Hospital  7=Non-governmental clinic or hospital  8=Other |
| **4.5** | Who attended the delivery?  **Circle all that are mentioned** | A=Doctor  B=Nurse  C=Midwife  D=Matrona  E=Relative  F=Friend  G=Other  H=No-one |
| **4.6** | What was used to cut the umbilical cord?  **Circle one** | 1=Razor blade  2=Household knife  3=Scissors  4=Other  5=Don’t know |
| **4.7** | Was it was sterilised?  **Circle one** | 0=No  1=Yes  2= Don’t know |
| **4.8** | If yes, how was it sterilised?  **Circle all that are mentioned**  **If the answer to 4.7 is no or don’t know, circle Not Applicable.** | A=Already sterilised (e.g. new blade from packet)  B=Alcohol  C=Heated  D=Washed with soap  E=Other  F=Don’t know  G=Not applicable |
| **4.9** | What was put on the umbilical cord after it was cut?  **Circle all that are mentioned** | A=Alcohol  B=Palm Oil  C=Siti Malagos  D=Bandage  E=Nothing  F=Other  G=Don’t know |
| **4.10** | How long after the baby was born did you first put the baby to the breast?  **If immediately, write 99.**  **Otherwise, write the number of hours or of days in the boxes. Circle to indicate if hours or days.** | 1=Hours  2=Days |
| **4.11** | At what age should a child first be given food other than breast milk: 1 month, 4 months, 6 months, 9 months, or 1 year?  **Circle one** | 1=1 month  2=4 months  3=6 months  4=9 months  5=1 year  6=Other  7=Don’t Know |
| **4.12** | There are some situations or signs that indicate that a pregnant woman should deliver in a clinic or a hospital. Do you know what any of these are?  **Circle all that are mentioned** | A=Very young age of mother  B=Old age of mother  C=Multiple births  D=Breach  E=Previous caesarean  F=High blood pressure  G=Anaemia  H=Oedema  I=Bleeding during pregnancy  J=Small size of the woman  K=Other  L=Don’t know |

**Explain that the following questions relate to vaccinations.**

| **Section 5: Vaccinations** | | | | |
| --- | --- | --- | --- | --- |
| **5.1** | | Ask for the name of the woman’s youngest child that is alive.  Ask when **NAME** was born.  **Write the month and year in the boxes** |  | |
| **5.2** | Can you show me a vaccination card where **NAME’S** vaccinations are written down?  **Circle one** | | | 0=No  1=Yes  2=Don’t Know |
| If yes, ask to see the card and write down whether the card shows that **NAME** has received:  **For each vaccination, tick yes, no, or the card cannot be read.**  **If there is no card available, go to Question 6.1.** | | | | |
| **5.3a** | BCG  **Circle one** | | | 0=No  1=Yes  2= Card can’t be read |
| **5.3b** | OPV1  **Circle one** | | | 0=No  1=Yes  2= Card can’t be read |
| **5.3c** | OPV2  **Circle one** | | | 0=No  1=Yes  2= Card can’t be read |
| **5.3d** | OPV3  **Circle one** | | | 0=No  1=Yes  2= Card can’t be read |
| **5.3e** | DPT1  **Circle one** | | | 0=No  1=Yes  2= Card can’t be read |
| **5.3f** | DPT2  **Circle one** | | | 0=No  1=Yes  2= Card can’t be read |
| **5.3g** | DPT3  **Circle one** | | | 0=No  1=Yes  2= Card can’t be read |
| **5.3h** | Measles  **Circle one** | | | 0=No  1=Yes  2= Card can’t be read |
| **5.4** | Where did **NAME** receive most of his or her vaccinations?  **Circle one** | | | 1=Hospital  2=Regional Clinic  3=NGO Clinic  4=Mobile Clinic  5=Other  6=Don’t know |

**Explain that the following questions are about accessing Health Care**

| **Section 6: Accessing Health Care** | | |
| --- | --- | --- |
| **6.1** | If your child (or one of your children) is seriously ill, can you decide by yourself whether or not the child should be taken for medical treatment? If she says the child is never seriously ill, then ask “If your child became seriously ill could you decide by yourself whether or not the child should be taken for medical treatment?”  **Circle one** | 0=No  1=Yes  2=Depends |
| Now I want to ask about medical treatment for yourself. Many different factors can prevent women from getting medical advice or treatment for themselves. When you are sick and want to get medical advice or treatment, is each of the following a big problem or not big problem?  **Read the list and, for each one, circle whether it is a big problem or not a big problem.** | | |
| **6.2a** | Knowing where to go  **Circle one** | 1=A big problem  2=Not a big problem |
| **6.2b** | Getting permission to go  **Circle one** | 1=A big problem  2=Not a big problem |
| **6.2c** | Getting money for needed treatment  **Circle one** | 1=A big problem  2=Not a big problem |
| **6.2d** | Distance to health facility  **Circle one** | 1=A big problem  2=Not a big problem |
| **6.2e** | Taking transport  **Circle one** | 1=A big problem  2=Not a big problem |
| **6.2f** | Going alone  **Circle one** | 1=A big problem  2=Not a big problem |
| **6.2g** | There may only be a male health provider  **Circle one** | 1=A big problem  2=Not a big problem |
| **6.2h** | There may not be anyone at the clinic or it will be closed  **Circle one** | 1=A big problem  2=Not a big problem |
| **6.2i** | The clinic will not be able to cure you  **Circle one** | 1=A big problem  2=Not a big problem |
| **6.2j** | The clinic will not have appropriate medicines  **Circle one** | 1=A big problem  2=Not a big problem |
| **6.3** | If your child is very sick, who would you normally seek help from first?  **Circle one** | 1=Another parent  2= Relative  3=Traditional Healer  4=ASC  5=Matrona  6=Clinic  7=Hospital  8=Other |
| **6.4** | If you need medicines for your children, where do you normally go to buy them?  **Circle all that are mentioned** | A=Local person  B=Relative  C=Pharmacy  D=USB  E=Local clinic / hospital  F=Other  G=Don’t know |
| **6.5** | How long does it take you to travel to this place to get medicines?  **Write the hours and minutes** | **________hours ________minutes** |
| **6.6** | Which medicines do you currently have in your house?  **Circle all that are mentioned** | A=ORS  B= Chloroquine  C= Fansidar  D=ACT  E= Paracetomol  F=Bactrim  G=Amoxycilin  H=Traditional Medicines  I=Other  J=Can’t identify  K=None  L=Don’t know |

**Now explain that you would like to observe some things in and around the house.**

| **Section 7: Observation Section** | | |
| --- | --- | --- |
| **7.1** | Ask to see where the household stores drinking water.  Check the following (observe and ask):  **Circle all that apply** | A=Clean  B=Covered  C=Dedicated ladle or cup to remove water that is not used to drink from directly  D=Ladle or cup to remove water that is use to drink from directly |
| **7.2** | Ask to see where the youngest child sleeps.  Do you see a bed net?  **Circle one** | 0=No  1=Yes |
| **7.3** | Ask how many bed nets there are in the house.  **Write the Number** |  |
| **7.4** | Ask when was the last time that the nets were impregnated?  **Circle one**  **If the house has no bed nets, circle Not Applicable** | 1=The net does not need impregnating yet.  2=The net is a long term one and does not need impregnating  3=Within the last six months  4=Within the last one year  5=Never  6=Other  7=Don’t Know  8=Not Applicable |
| **7.5** | Ask to see where she prepares food.  **Ask if they have a place for hand washing nearby.**  **Circle one** | 0=No  1=Yes |
| **7.6** | If the answer is yes, check if there is soap.  **If the answer to 7.5 is no, circle Not Applicable**  **Circle one** | 0=No  1=Yes  2=Not Applicable |
| **7.7** | Ask the respondent if the household has a latrine.  **Circle one** | 0=No  1=Yes |
| **7.8** | If yes, check the following:  **If the answer to 7.7 is no, circle Not Applicable**  **Circle all that apply.** | A=Clean  B=Covered with a lid  C=Has water for cleaning  D=Has a brush for cleaning  E=Has a place for hand-washing close by  F=Not Applicable |

**MAN’S SURVEY**

**Explain to the man that the first section relates to information about him.**

| **Section 2: Respondent Information** | | |
| --- | --- | --- |
| **2.1** | Sex  **Circle one** | 1=Male  2=Female |
| **2.2** | Age  **Ask how old he is in years.**  **Write the age in the boxes** |  |
| **2.3** | Ethnicity  **Ask him which ethnic group he belongs to.**  **Circle one** | 1=Balanta  2=Biafada  3=Fula  4=Nalu  5=Mandinka  6=Susu  7=Bijagos  8=Other |
| **2.4** | Schooling  **Ask if he has ever attended school.**  **Circle one** | 0-No  1=Yes |
| **2.5** | Education Level  **If yes, ask him what is the highest level he attended or is attending.**  **If the answer to 2.4 is no, circle Not Applicable**  **Circle one** | 1=Primary (Grade 1-6)  2=Secondary (Grade 7-9)  3=Higher (Grade 10-12)  4=Koranic School  5=Not applicable |
| **2.6** | Years of Schooling  **If yes, ask him how many years he attended school in total.**  **Write the number in the boxes**  **If the answer to 2.4 is no, Write N A for Not Applicable** |  |
| **2.7** | Literacy Level  **Ask him to read the sentence. If he cannot read the whole sentence, probe to read part of the sentence.**  **Circle one** | 1=Cannot read at all  2=Able to read only part of the sentence  3=Able to read the whole sentence  4=No appropriate language card available |

**Explain that the following questions relate to health knowledge.**

| **Section 3: Health Knowledge** | | |
| --- | --- | --- |
| **3.1** | If a child has diarrhoea, should he or she be given less to drink than usual, the same amount to drink as usual or more to drink than usual?  **Circle one** | 1=Less than usual  2=The same as usual  3=More than usual  4=Don’t know |
| **3.2** | Do you know any ways to prevent diarrhoea?  **Circle all that are mentioned** | A=Washing hands  B=Drinking clean water  C=Eating clean food  D=Using latrines  E= Putting lemon or lime in water  F=Other  G=Don’t know |
| **3.3** | Show him a packet or ORS.  **Ask, have you ever seen this before?**  **Circle one** | 0=No  1=Yes |
| **3.4** | Should a baby under the age of six months who has diarrhoea be given only breast milk, breast milk followed by ORS if necessary, only ORS, ORS followed by breast milk if necessary, nothing at all, or none of the above?  **Circle one** | 1=Only breast milk  2=Breast milk, followed by ORS if necessary  3=Only ORS  4=ORS, followed by breast milk if necessary  5=Nothing  6=None of the above  7=Don’t know |
| **3.5** | If a child is sick with fever, should he or she be wrapped up warmly and placed in a warm place, dressed as normal, dressed lightly and cooled with damp cloths, or none of the above?  **Circle one** | 1=Wrapped up warmly and placed in a warm place  2=Dressed as usual  3=Dressed lightly and cooled with damp cloths  4=None of the above  5=Don’t know |
| **3.6** | Have you ever heard of malaria?  **Circle one** | 0=No  1=Yes |
| **3.7** | If yes, how do people become infected with malaria?  **Circle all that are mentioned**  **If the answer to 3.6 is no, circle Not Applicable** | A=From a mosquito bite  B=Other  C=Don’t Know  D=Not applicable |
| **3.8** | How can people avoid malaria?  **Circle all that are mentioned**  **If the answer to 3.6 is no, circle Not Applicable** | A=Sleep under a bed net  B=Use insect repellents  C=Cover skin in the evening  D=Clear mosquito breeding sites (standing water; Vegetation)  E=Other  F=Don’t know  G=Not applicable |
| **3.9** | What medicines can be used to treat malaria?  **Circle all that are mentioned**  **If the answer to 3.6 is no, circle Not Applicable** | A=Chloroquine  B=Fansidar  C=ACT  D=Quinine  E=Paracetomol  F=Aspirin  G=Traditional medicines  H=Other  I=Don’t know  J=Not applicable |
| **3.10** | If you use chloroquine to treat a child with malaria should you give it once and then stop, keep giving it until the child feels better and then stop, give it for at least three days even if the child feels better, or something else?  **Circle one** | 1=Once, then stop.  2=Keep giving it until the child feels better, then stop.  3=Give it for at least three days even if the child feels better.  4=Something else  5=Don’t know  6=Not applicable |
| **3.11** | Have you ever heard of pneumonia?  **Circle one** | 0=No  1=Yes |
| **3.12** | If yes, what are the key symptoms of pneumonia?  **Circle all that are mentioned**  **If the answer to 3.11 is no, circle Not Applicable** | A=Cough  B=Breathing difficulties (fast or noisy breathing)  C=Indrawn chest  D=Other  E=Don’t know  F=Not applicable |
| **3.13** | If a child has pneumonia, what are the possible treatments that could cure the child?  **Circle all that are mentioned**  **If the answer to 3.11 is no, circle Not Applicable** | A=Antibiotics  B=Traditional medicines  C=Other  D=Don’t know  E=Not applicable |
| **3.14** | What is the best way to prevent measles?  **Circle all that are mentioned** | A=Vaccine  B=Keep children away from infected children  C=Other  D=Don’t know |
| **3.15** | Have you ever heard of HIV / AIDS?  **Circle one** | 0=No  1=Yes |

**Explain that the following questions are about accessing Health Care**

| **Section 6: Accessing Health Care** | | |
| --- | --- | --- |
| **6.3** | If your child is very sick, who would you normally seek help from first?  **Circle one** | 1=Another parent  2= Relative  3=Traditional Healer  4=ASC  5=Matrona  6=Clinic  7=Hospital  8=Other |
| **6.4** | If you need medicines for your children, where do you normally go to buy them?  **Circle all that are mentioned** | A=Local person  B=Relative  C=Pharmacy  D=USB  E=Local clinic / hospital  F=Other  G=Don’t know |
| **6.5** | How long does it take you to travel to this place to get medicines?  **Write the hours and minutes** | **________hours ________minutes** |
| **6.6** | Which medicines do you currently have in your house?  **Circle all that are mentioned** | A=ORS  B= Chloroquine  C= Fansidar  D=ACT  E= Paracetomol  F=Bactrim  G=Amoxycilin  H=Traditional Medicines  I=Other  J=Can’t identify  K=None  L=Don’t know |

Time Finished
